# A Cross-Sectional Study of Celiac Disease Awareness in the Food Industry in the Western Region of Saudi Arabia

**DOI:** 10.7759/cureus.25613

**Published:** 2022-06-02

**Authors:** Abdullah A Khafagy, Wadah K Qari, Suhail S Filimban, Abdulhafiz M Bahalaq, Adeeb A Bulkhi

**Affiliations:** 1 Department of Community Medicine and Pilgrims Healthcare, Umm Al-Qura University, Makkah, SAU; 2 Department of Internal Medicine, Umm Al-Qura University, Makkah, SAU

**Keywords:** gluten sensitivity, restaurant, food handling, gluten-free diet, celiac disease

## Abstract

Background

The only treatment available for celiac disease (CD), an autoimmune disease, is a gluten-free diet. Restaurant personnel have major roles in understanding the possible risks to consumers with CD, ensuring the availability of and preparing gluten-free foods. We attempted to evaluate the awareness and knowledge of CD among chefs, cooks, restaurant personnel, and owners and assess the availability of gluten-free diet options in the western region of Saudi Arabia.

Methods

A cross-sectional, questionnaire-based study was conducted in 126 restaurants based in Makkah al-Mukarramah and Jeddah cities. The chefs and owners of the restaurants were interviewed face-to-face to collect data knowledge about CD, gluten sensitivity, food containing gluten, serving gluten-free food, intention to add gluten-free options in the future, and circumstances related to serving gluten-free food.

Result

Our study showed that 17.5% and 51.6% of the participants had heard about CD and gluten sensitivity, respectively, and 34.1% checked a right answer of gluten-containing food with a mean of 0.68 (±1.02). About 17.5% of the participating restaurants serve gluten-free meal options (mean: 0.63±1.57), 14.7% had protocols for the preparation of gluten-free food, 7.1% displayed signs or notices that they sell gluten-free products, and 50.8% disclosed an intention to add gluten-free options in the future. Furthermore, 82.5% of gluten-free options were more expensive. Education level, being a trained chef, and experience years were significantly associated with awareness about CD or gluten sensitivity (p<0.05).

Conclusion

There is a general lack of awareness of CD, and most restaurants lack gluten-free options. We recommend adding more gluten-free food options for patients with CD.

## Introduction

Celiac disease (CD) is an autoimmune disease characterized by inflammation of the mucosa of the small bowel triggered by gluten. The prevalence of CD varies greatly worldwide; an estimated 1% of many populations are affected [1]. Although the exact mode of inheritance is unknown, the HLA-DQ2 and HLA-DQ8 genes were found to be associated with the disease [1]. Most cases are asymptomatic; in symptomatic cases, the most common symptoms are tiredness, malaise, diarrhea, abdominal pain, and weight loss [1]. It is also associated with anemia due to malabsorption.

People with CD have twice the risk of developing coronary artery disease and four times greater risk of developing small bowel cancers [2]. Untreated CD can lead to the development of other autoimmune disorders such as type I diabetes and multiple sclerosis. Additionally, undiagnosed or untreated CD can lead to long-term health conditions such as iron deficiency anemia, early-onset osteoporosis or osteopenia, infertility and miscarriage, lactose intolerance, and vitamin and mineral deficiencies, whereas early diagnosis lowers the chances of developing other conditions [2].

In Saudi Arabia, the estimated prevalence of CD among children is 1.5%, a rate that is at least twice the average prevalence rate in Europe and North America [3]. Several studies have estimated the prevalence of CD in Saudi Arabia in different age groups. A study on 1,141 asymptomatic children of age between 6 and 18 years in the eastern province of Saudi Arabia estimated the prevalence to be 1% [4], while another study on 1,167 adolescent students estimated the prevalence to be 1.8%-3.2% [5]. Furthermore, a study on adults estimated the prevalence to be 1.5% [3]. Although the estimated prevalence varies between age groups in different studies, it is commonly higher than the average prevalence (1%), indicating a need for gluten-free food availability.

Currently, the only treatment for CD is lifelong adherence to a strict gluten-free diet. Due to the limitations on consumable food items, individuals suffering from CD face challenges in eating out at restaurants and other food establishments, fearing accidental exposure to gluten food and/or the unavailability of gluten-free food. The restaurant personnel has an important role in understanding the possible risks to these consumers.

There are several attempts to evaluate knowledge of CD among the public, chefs, cooks, restaurant personnel, and physician across the globe, revealing the different levels of knowledge and awareness among the stakeholders. A study was conducted in New Zealand to investigate awareness about CD among the public, chefs, and cooks. The study examined the knowledge of CD and gluten-free food preparation among chefs and cooks and revealed that almost all the participants were aware of CD despite a lack of any formal training [6]. A questionnaire-based study in the UK determined the change in the level of awareness about gluten-related disorders among the general public and chefs between 2003 and 2013. The study reported a marked increase in both the public’s and chefs’ awareness of gluten-related disorders during the period [7,8].

A cross-sectional survey to assess knowledge of CD among physicians in primary, secondary, and tertiary care public and private hospitals in Riyadh, Saudi Arabia, revealed poor knowledge of CD among a significant number of physicians [9]. The poor knowledge level can potentially lead to delays in diagnosis.

A systematic review of published research on the prevalence of food allergy and CD knowledge, practices, and training among restaurant and food service personnel demonstrated a lack of global research on the subject as most of the relevant studies were conducted in either the USA (50%) or UK (26%) [10]. The systemic review found that there is comparatively less research focused on knowledge and practices related to CD compared to food allergy [10]. Additionally, they reported a lack of reporting gluten-free food availability on menus or other documentation. Individuals with CD often rely on written information provided to make informed decisions about their purchases and dining.

Despite the knowledge of gluten-related disorders and the importance of the gluten-free diet for managing the disease among the general public, chefs, and cooks, there is still limited availability of gluten-free foods, and the food is comparatively expensive [11]. The knowledge, availability, and costs of the gluten-free diet are vital in ensuring dietary compliance in CD.

There is a paucity of studies in Saudi Arabia that evaluated the level of knowledge of chefs and restaurant owners about CD and gluten-free diet, availability of gluten-free food options, and its prices compared to standard counterparts. Therefore, we conducted this study to evaluate the knowledge of chefs and restaurant owners about CD and assess the availability of gluten-free diet options in the western region of Saudi Arabia.

## Materials and methods

This was a cross-sectional study assessing the awareness of CD among chefs and restaurant owners in the western region of Saudi Arabia and the availability of gluten-free diet options in these restaurants. We interviewed restaurant chefs and owners to complete the questionnaire (Appendix 1). A total of 126 restaurants were selected randomly based on the geographical distribution of the restaurants in the city. We used the Foursquare application restaurant list for restaurants in Makkah al-Mukarramah city and Jeddah Municipality website for restaurants in Jeddah city. The restaurants were selected using Google random number generator. We visited the restaurants for face-to-face interviews with the chefs or the owners. If we were not able to complete the questionnaire during the first attempt due to the nonavailability of the chef/owner, a follow-up phone was attempted to complete the questionnaire. If the restaurants did not complete the questionnaire after the second attempt, it was counted as a nonresponse.

The inclusion criteria were restaurants operating in Makkah al-Mukarramah city or Jeddah city, including fancy restaurants, fast food, cafes, healthy restaurants, and bakeries with chefs or owners able to communicate in Arabic or English. The exclusion criteria were traditional restaurants.

A predesigned questionnaire was used to collect data on knowledge about CD, gluten sensitivity, food containing gluten, serving gluten-free food and intention to add gluten-free options in the future, and circumstances related to serving gluten-free food, along with data on the participant’s demographics and work information.

Ethical considerations

Ethical approval for the study was obtained from the Biomedical Research Ethics Committee of Umm Al-Qura University, College of Medicine.

Statistical analysis

Data were analyzed using the STATA/IC software version v16.1 (StataCorp LLC, TX, USA). Qualitative data were expressed as numbers and percentages. The chi-squared test (χ2) was applied to test the relationship between variables. Quantitative data were presented as means and standard deviations (mean±SD), and the Mann-Whitney test was applied for analyzing nonparametric variables. A p-value of <0.05 was considered statistically significant.

## Results

The basic characteristics of the restaurants included in the study are summarized in Table 1. Most of the questionnaires were answered by the chef (86.5%). Of the total participating chefs and owners, 37.3% had a bachelor’s degree and 74.6% had studied culinary art, and their mean years of experience was 9.88 (±7.4) years.

**Table 1:** Distribution of the studied participants according to their demographics, work information, and circumstances related to serving gluten-free food (N=126) SD: standard deviation

Variable	No. (%)
City	
Makkah	59 (46.8)
Jeddah	67 (53.2)
Type of restaurant	
Fast food	20 (15.9)
Fast casual	9 (7.1)
Casual dining	22 (17.5)
Fine dining	28 (22.2)
Cafes	24 (19)
Healthy diet	1 (0.8)
Bakery	22 (17.5)
Type of meal	
American	23 (18.3)
Asian	4 (3.2)
Seafood	5 (4)
Italian	9 (7.1)
French	2 (1.6)
Diner	3 (2.4)
Dessert	39 (31)
Arabic	19 (15.1)
Others	22 (17.5)
Average meal pricing	
Less than 50 SR	77 (61.1)
50-150 SR	43 (34.1)
More than 150 SR	6 (4.8)
Number of branches	
1	54 (42.9)
2-9	44 (34.9)
More than 10	28 (22.2)
Questionnaire answered by	
Owner	17 (13.5)
Chef	109 (86.5)
Chef or owner gender	
Male	123 (97.6)
Female	3 (2.4)
Chef or owner age	
Less than 35	72 (57.1)
Older than 35	54 (42.9)
Chef or owner nationality	
Saudi	34 (27)
Non-Saudi	92 (73)
Chef or owner education level	
Non-formal education	6 (4.8)
Middle school	13 (10.3)
High school	37 (29.4)
Bachelor	47 (37.3)
Diploma	15 (11.9)
Master	6 (4.8)
PhD or doctoral	2 (1.6)
Field of study related to	
Culinary art	32 (25.4)
None of the above	94 (74.6)
Trained chef/cook	
Yes	79 (62.7)
No	47 (37.3)
Experience (years) (SD)	9.88 ± 7.4

Our study showed that only 17.5% of the participants had heard about CD, 51.6% heard about gluten sensitivity, and 34.1% checked a right answer of gluten-containing food with a mean number of right answers of 0.68 (±1.02) (Table 2). About 17.5% of the participating restaurants serve gluten-free meal options with a mean number of options of 0.63 (±1.57). The prices of 82.5% of the gluten-free options were more expensive compared to non-gluten-free options. A low number of restaurants (14.7%) had protocols for the preparation of gluten-free foods and displayed signs or notices that they sell gluten-free products (7.1%). Nearly half of the participating restaurants (50.8%) disclosed an intention to add gluten-free options in the future.

**Table 2:** Distribution of the studied participants according to knowledge about celiac disease, gluten sensitivity, food containing gluten, serving gluten-free food, and intention to add gluten-free options in the future (N=126)

Variable	No. (%)
Hear about celiac disease	
Yes	22 (17.5)
No	104 (82.5)
Heard about gluten sensitivity	
Yes	65 (51.6)
No	61 (48.4)
Identifying items that contain gluten	
Correctly	43 (34.1)
Wrongly	83 (65.9)
Number of right answers (mean±SD)	0.68±1.02
Serve any gluten-free meal options	
Yes	22(17.5)
No	104 (82.5)
Number of available options	0.63±1.57
Price of gluten-free options on average compared to non-gluten-free options	
Cheaper	17 (13.5)
About the same	5 (4)
More expensive	104 (82.5)
Any protocol for the preparation of gluten-free food	
Yes	16 (14.7)
No	6 (4.8)
Not relevant	104 (82.5)
Any displayed signs or notices that you sell gluten-free products	
Yes	9 (7.1)
No	117 (92.9)
Any intention to add any gluten-free options in the future	
Yes	64 (50.8)
No	62 (49.2)

The awareness and knowledge of CD were significantly higher among participants from Jeddah, chefs/owners of restaurants serving fine dining meals, having a meal price of more than 150 SR, and a chef or owner with an age of more than 35 years (p<0.05) (Table 3). At the same time, chefs with a bachelor’s degree of education, who studied culinary art, and who were trained chefs with longer mean years of experience had a significantly higher percentage of those who heard about CD (p<0.05). The participants who had heard about gluten sensitivity, who checked the right answers for gluten-free food or a higher number of right answers, who serve any gluten-free meal options, and had a higher mean number of served options had a significantly higher percentage of those who heard about CD (p<0.05) (Table 4). Furthermore, the participants who reported that the price of gluten-free options compared to non-gluten-free options was about the same, who had a protocol for the preparation of gluten-free food, who displayed signs or notices that gluten-free products are available, and who had an intention to add any gluten-free options in the future had a significantly higher percentage of those who heard about CD (p<0.05).

**Table 3:** Relationship between hearing about celiac diseases and participants’ demographics, work information, and circumstances related to serving gluten-free food (N=126) SD: standard deviation

Variable	Heard about celiac disease		P-value
	Yes (number (%))	No (number (%))	
City			
Makkah	5 (8.5)	54 (91.5)	0.013
Jeddah	17 (25.4)	50 (74.6)	
Type of restaurant			
Fast food	1 (5)	19 (95)	<0.001
Fast casual	1 (11.1)	8 (88.9)	
Casual dining	0 (0)	22 (100)	
Fine dining	14 (50)	14 (50)	
Cafes	2 (8.3)	22 (91.7)	
Healthy diet	0 (0)	1 (100)	
Bakery	4 (18.2)	18 (81.8)	
Type of meal			
American	3 (13)	20 (87)	0.457
Asian	1 (25)	3 (75)	
Seafood	1 (20)	4 (80)	
Italian	1 (11.1)	8 (88.9)	
French	1 (50)	1 (50)	
Diner	2 (66.7)	1 (33.3)	
Dessert	6 (15.4)	33 (84.6)	
Arabic	4 (21.1)	15 (78.9)	
Others	3 (13.6)	19 (86.4)	
Average meal pricing			
Less than 50 SR	7 (9.1)	70 (90.9)	0.008
50-150 SR	13 (20.2)	30 (69.8)	
More than 150 SR	2 (33.3)	4 (66.7)	
Number of branches			
1	11 (20.4)	43 (79.6)	0.544
2-9	8 (18.2)	36 (81.8)	
More than 10	3 (10.7)	25 (89.3)	
Questionnaire answered by			
Owner	2 (11.9)	15 (88.2)	0.506
Chef	20 (18.3)	89 (81.7)	
Chef or owner gender			
Male	21 (17.1)	102 (82.9)	0.464
Female	1 (33.3)	2 (66.7)	
Chef or owner age			
Less than 35	5 (6.9)	67 (93.1)	<0.001
Older than 35	17 (31.5)	37 (68.5)	
Chef or owner nationality			
Saudi	5 (14.7)	29 (85.3)	0.621
Non-Saudi	17 (18.5)	75 (81.5)	
Chef or owner education level			
Non-formal education	0 (0)	6 (100)	0.031
Middle school	0 (0)	13 (100)	
High school	4 (10.8)	33 (89.2)	
Bachelor	14 (29.8)	33 (70.2)	
Diploma	1 (6.7)	14 (93.3)	
Master	2 (33.3)	4 (66.7)	
PhD or doctoral	1 (50)	1 (50)	
Field of study related to			
Culinary art	16 (50)	16 (50)	<0.001
None of the above	6 (6.4)	88 (93.6)	
Trained chef/cook			
Yes	21 (26.6)	58 (73.4)	<0.001
No	1 (2.1)	46 (97.9)	
Experience (years) (SD)	14.55±5.66	8.89±7.37	<0.001

**Table 4:** Relationship between hearing about celiac diseases and knowledge about celiac disease, gluten sensitivity, food containing gluten, serving gluten-free food, and intention to add gluten-free options in the future (N=126) SD: standard deviation

Variable	Heard about celiac disease		P-value
	Yes (number (%))	Yes (number (%))	
Heard about gluten sensitivity			
Yes	22 (33.8)	43 (66.2)	<0.001
No	0 (0)	61 (100)	
Identifying items that contain gluten			
Correctly	20 (46.5)	23 (53.5)	<0.001
Wrongly	2 (2.4)	81 (97.6)	
Number of right answers (mean±SD)	1.9±0.75	0.42±0.87	<0.001
Serve any gluten-free meal options			
Yes	13 (59.1)	9 (40.9)	<0.001
No	9 (8.7)	95 (91.3)	
Number of available options (mean±SD)	1.95±2.17	0.35±1.26	
Price of gluten-free options on average compared to non-gluten-free options			
Cheaper	8 (47.4)	9 (52.9)	<0.001
About the same	5 (100)	0 (0)	
More expensive	9 (8.7)	95 (91.3)	
Any protocol for the preparation of gluten-free food			
Yes	10 (62.5)	6 (37.5)	<0.001
No	3 (50)	3 (50)	
Not relevant	9 (8.7)	95 (91.3)	
Any displayed signs or notices that you sell gluten-free products			
Yes	5 (55.6)	4 (44.4)	0.002
No	17 (14.5)	100 (85.5)	
Any intention to add any gluten-free options in the future			
Yes	19 (29.7)	45 (70.3)	<0.001
No	3 (4.8)	59 (95.2)	

The participants from Jeddah city, participants serving fine dining meals, and participating restaurants that had average meal pricing of more than 150 SR had a significantly higher percentage of chefs/owners who heard about gluten sensitivity (p<0.05). A significantly higher percentage of participants who heard about gluten sensitivity were owners with a master’s degree of education, had studied culinary art, were trained chefs/cooks, and had a longer mean of years of experience (p<0.05) (Table 5).

**Table 5:** Relationship between hearing about gluten sensitivity and participants’ demographics, work information, and circumstances related to serving gluten-free food (N=126) SD: standard deviation

Variable	Heard about gluten sensitivity		P-value
	Yes (number (%))	No (number (%))	
City			
Makkah	24 (40.7)	35 (59.3)	0.021
Jeddah	41 (61.2)	26 (38.8)	
Type of restaurant			
Fast food	7 (35)	13 (65)	0.008
Fast casual	5 (55.6)	4 (44.4)	
Casual dining	7 (31.8)	15 (68.2)	
Fine dining	23 (82.1)	5 (17.9)	
Cafes	12 (50)	12 (50)	
Healthy diet	0 (0)	1 (100)	
Bakery	11 (50)	11 (50)	
Type of meal			
American	15 (65.2)	8 (34.8)	0.405
Asian	2 (50)	2 (50)	
Seafood	1 (20)	4 (80)	
Italian	4 (44.4)	5 (55.6)	
French	2 (100)	0 (0)	
Diner	2 (66.7)	1 (33.3)	
Dessert	16 (41)	23 (59)	
Arabic	10 (52.6)	9 (47.4)	
Others	13 (59.1)	9 (40.9)	
Average meal pricing			
Less than 50 SR	30 (39)	47 (61)	0.001
50-150 SR	29 (67.4)	14 (32.6)	
More than 150 SR	6 (100)	0 (0)	
Number of branches			
1	34 (63)	20 (37)	0.076
2-9	20 (45.5)	24 (54.5)	
More than 10	11 (39.3)	17 (60.7)	
Questionnaire answered by			
Owner	10 (58.8)	7 (41.2)	0.521
Chef	55 (50.5)	54 (49.5)	
Chef or owner gender			
Male	63 (51.2)	60 (48.8)	0.597
Female	2 (66.7)	1 (33.3)	
Chef or owner age			
Less than 35	31 (43.1)	41 (56.7)	0.027
Older than 35	34 (63)	20 (37)	
Chef or owner nationality			
Saudi	17 (50)	17 (50)	0.828
Non-Saudi	48 (52.2)	44 (47.8)	
Chef or owner education level			
Non-formal education	0 (0)	6 (100)	<0.001
Middle school	3 (23.1)	10 (76.9)	
High school	13 (35.1)	24 (64.9)	
Bachelor	33 (70.2)	14 (29.8)	
Diploma	10 (66.7)	5 (33.3)	
Master	5 (83.3)	1 (16.7)	
PhD or doctoral	1 (50)	1 (50)	
Field of study related to			
Culinary art	27 (84.4)	5 (15.6)	<0.001
None of the above	38 (40.4)	56 (59.6)	
Trained chef/cook			
Yes	59 (62)	30 (38)	<0.001
No	16 (34)	31 (66)	
Experience (years) (SD)	11.42±7.86	8.23±6.55	<0.001

The study also illustrated that participants who answered right about gluten-free food and had a high mean number of right answers and those serving gluten-free meal options with a higher mean number of options had a significantly higher percentage of those who heard about gluten sensitivity (p<0.05) (Table 6). Participants who reported that the average price of the gluten-free option was cheaper or about the same compared to the non-gluten-free option, who had displayed signs or notices about gluten-free products’ availability, and had the intentions to add any gluten-free options in the future had a significantly higher percentage of those who heard about gluten sensitivity (p<0.05).

**Figure 1:**
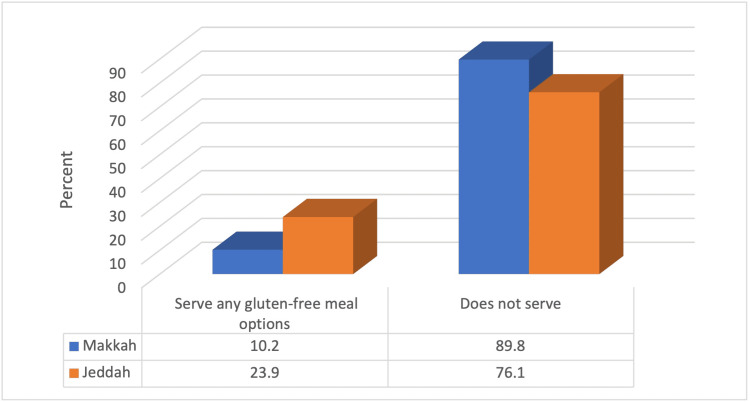
Relationship between serving gluten-free food and city (N=126) χ2=4.09 p=0.043

We found that significantly more restaurants from Jeddah city serve gluten-free meal options compared to those from Makkah City (p<0.05) (Figure 1).

The study demonstrated that participants serving fine dining meals, had an average meal price of more than 150 SR, with a chef or owner >35 years of age, had a bachelor’s degree of education, studied culinary art, were trained chefs/cooks, and have a longer mean of years of experience had a significantly higher percentage of those who serve gluten-free food (p<0.05) (Table 7).

**Table 6:** Relationship between serving gluten-free food and work circumstances, food containing gluten, and intention to add gluten-free options in the future (N=126) SD: standard deviation

Variable	Serve any gluten-free food		P-value
	Yes (number (%))	No (number (%))	
Type of restaurant			
Fast food	0 (0)	20 (100)	0.012
Fast casual	2 (22.2)	7 (77.8)	
Casual dining	1 (4.5)	21 (95.5)	
Fine dining	11 (39.3)	17 (60.7)	
Cafes	4 (16.7)	20 (83.3)	
Healthy diet	0 (0)	1 (100)	
Bakery	4 (18.2)	18 (81.8)	
Average meal pricing			
Less than 50 SR	9 (11.70)	68 (88.3)	<0.001
50-150 SR	8 (18.6)	35 (81.4)	
More than 150 SR	5 (83.3)	1 (16.7)	
Chef or owner age			
Less than 35	7 (9.7)	65 (90.3)	0.008
Older than 35	15 (27.8)	39 (72.2)	
Chef or owner education level			
Non-formal education	0 (0)	6 (100)	0.003
Middle school	0 (0)	13 (100)	
High school	4 (10.8)	33 (89.2)	
Bachelor	15 (31.9)	32 (68.1)	
Diploma	0 (0)	15 (100)	
Master	3 (50)	3 (50)	
PhD or doctoral	0 (0)	2 (100)	
Field of study related to			
Culinary art	15 (46.9)	17 (53.1)	<0.001
None of the above	7 (7.4)	87 (92.6)	
Trained chef/cook			
Yes	21 (26.6)	58 (73.4)	<0.001
No	1 (2.1)	46 (97.9)	
Experience (years) (SD)	14.32±8.65	8.94±6.79	0.003
Identifying items that contain gluten			
Correctly	16 (37.2)	27 (62.8)	
Wrongly	6 (7.2)	77 (92.8)	
Number of right answers (mean±SD)	1.45±0.01	0.51±0.95	
Number of available options	3.63±1.81	0.001±0.001	<0.001
Price of gluten-free options on average compared to non-gluten-free options			
Cheaper	17 (100)	0 (0)	<0.001
About the same	5 (100)	0 (0)	
More expensive	0 (0)	104 (100)	
Any protocol for the preparation of gluten-free food			
Yes	16 (100)	0 (0)	<0.001
No	6 (100)	0 (0)	
Not relevant	0 (0)	104 (100)	
Any displayed signs or notices that you sell gluten-free products			
Yes	9 (100)	0 (0)	<0.001
No	13 (11.1)	104 (88.9)	
Any intention to add any gluten-free options in the future			
Yes	19 (29.7)	45 (70.3)	<0.001
No	3 (4.8)	59 (95.2)	

## Discussion

The only treatment for CD is lifelong compliance with the gluten-free diet. This imposes challenges in dining out to individuals with CD, impacting their quality of life. The impact is intensified by the scarce availability and cost of gluten-free food options and the knowledge level of CD among the restaurant personnel. Earlier CD was considered a very rare children’s disease, and awareness about dietary restrictions on gluten was relatively low among medical professionals and the general public. Time and new facts from research have increased awareness about CD [12].

After conducting face-to-face interviews in 126 restaurants, we found that about half (51.6%) of the chefs and owners know about gluten sensitivity and only 17.5% know about CD. This indicates a lack of awareness among restaurants about an important disease that affects our population. The lack of awareness was higher among untrained chefs. In contrast, a significantly higher percentage of chefs and owners with bachelor’s or master’s degrees had heard about CD and gluten sensitivity. There was a significant association between the level of education, being a trained chef, and the years of experience and hearing about CD or gluten sensitivity. A study conducted in the United Kingdom demonstrated that the one-hour lecture on knowledge level about food allergies and gluten-free diet improved the participants’ knowledge and ability to identify the allergen [7,8].

A systematic review was conducted in 38 relevant clinical studies on the knowledge, practices, and training of restaurant and food service personnel regarding food allergies and CD [10]. The systemic review highlighted the lower amount of research for CD; however, the awareness about CD and a gluten-free diet has been increasing. Consistent with our study, the systemic review also identified gaps in the reporting of the availability of gluten-free food on menus and other documents.

A United Kingdom-based study conducted in 2003 and a 10-year follow-up in 2013 also demonstrated an increase in awareness about CD among the public and chefs; chefs were more knowledgeable [7,8]. Our study did not include the general public, so such a comparison cannot be made.

Among 126 restaurants, only 22 provide gluten-free options, and they are fine dining restaurants. The average meal pricing of gluten-free options of less than 50 SR was available only in 11.7% of the restaurants compared to 88.3% of non-gluten-free options. The limited availability of gluten-free foods and comparatively higher price may affect compliance with a gluten-free diet, increasing the risk of complications [11].

A survey conducted in Turkey that evaluated public awareness and sensitivity toward CD found that the public’s attention to the existence of gluten-free products in markets was directly significant to their education level (p=0.000), and half of the subjects (49.3%) were unaware of GF products. Similarly, another study reported that celiac patients have moderate to high difficulty in reaching gluten-free products [13]. These results are comparable to our study results.

Considering the high prevalence of CD in Saudi Arabia, the higher availability of gluten-free food options at a comparable cost to standard food items needs to be improved. The awareness about CD and gluten-free diet among chefs, owners, and restaurant personnel should be enhanced. Disordered eating behaviors (DEBs) prevail in celiac individuals of all age groups, which presents a risk of developing eating disorders [14].

A US study assessing the knowledge of CD and gluten sensitivity, as well as dining habits among the public and chefs, concluded that individuals with CD avoid restaurants and eat outside the home less frequently than the general public [15].

In contrast to our study results, a study conducted in New Zealand reported a high level of knowledge among half of the participating restaurant personnel despite a lack of formal training [6]. At the same time, the study concluded with a warning that awareness does not necessarily translate to standard operating procedures and policies to prepare gluten-free food [6]. This highlights the key area for gluten-free diet compliance.

In Saudi Arabia, there is no data available about the level of knowledge among chefs and restaurant owners about CD, gluten-free diet, availability of options of a gluten-free diet in restaurants, and their prices compared to standard counterparts. This is the first study to focus on this important aspect of CD. Our study focused on the unaddressed issues about CD in Saudi Arabia. This could be counted as the first step toward creating awareness about the issue. We conducted all interviews in person but did not prearrange an appointment.

Our study was limited to restaurants in Makkah and Jeddah cities due to limited resources. The database we used for restaurants in Makkah was the Foursquare website, which is not a formal reference list and is not inclusive of all the restaurants in Makkah. We could not complete all the restaurants on the list because of the COVID-19 crisis and extended the duration of the study to two years for data collection. Since some of the restaurants on our list were closed, we had to replace them with other restaurants. For better outcomes in future research, a bigger sample and a wider geographical region should be considered.

Our study was focused on restaurants and restaurant personnel, but a wider population study to evaluate the general public awareness, availability of packed gluten-free food items in stores, and labeling standards for gluten-free food is equally important. Gluten-free food items should be categorized based on the quantity of gluten content and a group of people intended to benefit, e.g., gluten-intolerant/celiac individuals and healthy individuals, for nutrition purposes. Legislation to identify the tolerable gluten quantity in the gluten-free food for celiac individuals, labeling standards for gluten-free food, and pricing are advised.

## Conclusions

The study evaluated the level of knowledge about celiac disease among chefs and restaurant owners in two cities in Saudi Arabia and the availability of gluten-free diet options. We found that there is a general lack of awareness of CD, very limited availability of gluten-free options, and a lack of display about their availability. Lack of awareness was directly associated with the level of education and training.

A larger study to accommodate a larger population and different stakeholders at the national level, training programs to improve awareness about CD and gluten-free diet, and comprehensive legislation to cover labeling standards, categorization, and cost of gluten-free food options are recommended.

## Appendices

Questionnaire form

Restaurant name:

City:

Region:

District:

Type of restaurant:

1. Fast food

2. Fast causal

3. Casual dining

4. Fine dining

5. Cafes

6. Healthy diet/subscription

7. Bakery

Type of meal:

1. American

2. Asian

3. Seafood

4. Italian

5. French

6. African

7. Mexican

8. Dessert

9. Others

Average meal pricing:

1. Less than 50 SR

2. 50-150 SR

3. More than 150 SR

Number of branches:

1. 1

2. 2-9

3. More than 10

Questionnaire answered by:

1. Owner

2. Chef

Chef or owner details:

Gender:

Age:

1. Less than 35

2. More than 35

Nationality:

1. Saudi

2. Egyptian

3. Syrian

4. Yemenis

5. Indonesian

6. Philippines

7. Indian

8. Pakistani

9. Bangladesh

10. Others

Educational level:

1. Non-formal education

2. Middle school

3. High school

4. Bachelor

5. Master

6. Diploma

7. PhD or doctoral

Field of study related to:

1. Culinary art

2. Nutrition

3. None of the above

Are you a trained chef/cook?

1. Yes

2. No

Qualification year:

Years of experience:

Have you heard about celiac disease?

1. Yes

2. No

Have you heard about gluten sensitivity?

1. Yes

2. No

Check all items that contain gluten (can select multiple):

1. Chicken

2. Bread

3. Pasta

4. Peanut butter

5. Salt

6. Oil

7. Rice

Do you serve any gluten-free meal options?

1. Yes

2. No

Number of available options:

1. 1

2. 2

3. 3

4. 4

5. 5 or more

Price of gluten-free options on average compared to non-gluten-free options:

1. Cheaper

2. About the same

3. More expensive

Do you have any protocol for the preparation of gluten-free food?

1. Yes

2. No

If yes, mention the protocol (separating cooking services and equipment, choice of ingredients for a gluten-free diet, etc.):

Any displayed signs or notices selling gluten-free products?

1. Yes

2. No

Do you have any intention to add any gluten-free options in the future?

1. Yes

2. No
